# On reproduction in red algae: further research needed at the molecular level

**DOI:** 10.3389/fpls.2015.00093

**Published:** 2015-02-23

**Authors:** Pilar García-Jiménez, Rafael R. Robaina

**Affiliations:** Departamento de Biología, Universidad de Las Palmas de Gran Canaria, Las Palmas de Gran Canaria, Spain

**Keywords:** Rhodophyta, seaweeds, reproduction, light, hormones, photoreceptors, signaling

## Abstract

Multicellular red algae (Rhodophyta) have some of the most complex life cycles known in living organisms. Economically valuable seaweeds, such as phycocolloid producers, have a triphasic (gametophyte, carposporophyte, and tetrasporophyte) life cycle, not to mention the intricate alternation of generations in the edible “sushi-alga” nori. It is a well-known fact that reproductive processes are controlled by one or more abiotic factor(s), including day length, light quality, temperature, and nutrients. Likewise, endogenous chemical factors such as plant growth regulators have been reported to affect reproductive events in some red seaweeds. Still, in the genomic era and given the high throughput techniques at our disposal, our knowledge about the endogenous molecular machinery lags far behind that of higher plants. Any potential effective control of the reproductive process will entail revisiting most of these results and facts to answer basic biological questions as yet unresolved. Recent results have shed light on the involvement of several genes in red alga reproductive events. In addition, a working species characterized by a simple filamentous architecture, easy cultivation, and accessible genomes may also facilitate our task.

## RHODOPHYTA AND REPRODUCTION. MERGING APPLIED AND FUNDAMENTAL KNOWLEDGE INTEREST

As recently reviewed (Rebours et al., 2014), the seaweed industry produces some 10 billion US$. Among the species exploited, the red seaweeds (Rhodophyta) *Eucheuma/Kappaphycus*, *Porphyra*, and *Gracilaria* occupy a leading position (*Kappaphycus* alone generates 1.3 billion US$ while the nori market is estimated at 1.5 billion US$). Nevertheless, this industry mostly relies on the exploitation of natural populations or primitive aquaculture methods, its expansion being restricted by the lack of technical and knowledge advances. An example of this can be seen in the absence of control of reproductive traits that would allow us to increase production, strain selection and breeding, a major step forward which has been achieved in land plant culture.

Rhodophyta are classified as Archaeplastida, along with glaucophytes and Viridiplantae (land plants and green algae) from which they diverge 1,500 Mya (Yoon et al., 2004). Like other algal groups, red algae comprise a myriad of species with different types of body architecture, ranging from the unicellular and filamentous to the blade or pseudo-parenchymatous as the most complex, particularly in the case of industrially valuable seaweeds (Cole and Sheath, 1995). Their extremely complex life cycles include the transition from unicellularity to complex multicellular bodies, the underlying molecular bases of which are virtually unknown. The diploid “conchocelis” and the meiotic-derived conchospores sustain the industry of “sushi,” since the tiny unicellular meiospore, the conchospore, grows and develops into the haploid leafy thallus, which is the edible phase (Drew, 1949, 1954).

**FIGURE 1:**
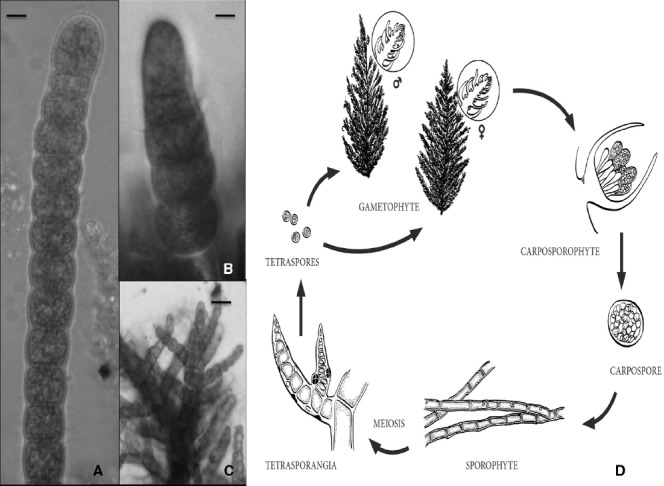
**(A)** Apical portion of a sporophyte branch of *Bonnemaisonia hamifera*, scale bar = 120 μm. **(B)** Putative gametophyte sporeling, scale bar = 25 μm. **(C)** Apex of a immature gametophytic thalli, scale bar = 100 μm. **(D)** Diagram of putative trigenetic life cycle in the red alga *Bonnemaisonia* comprising the gametophytes (haploids), the so-called carposporophyte that develops on the female gametophyte after fertilization, and the sporophyte (diploid) (adapted from *B. geniculata* in Shevlin and Polanshek, 1978. Not a scale).

Other economically valuable red algae, such as the producers of the phycocolloids agar or carrageen, have a trigenetic life cycle in which the haploid unicellular meiotic tetraspores germinate to produce both a male and a female multicellular gametophyte thalli (Cole and Sheath, 1995). Fertilization occurs when a spermatium fertilizes a carpogonium on the female gametophyte. The fertilized carpogonium develops into a structure called the cystocarp (diploid) after complex cell differentiation events, leading to the accumulation of mitotic diploid carpospores. Eventually, diploid carpospores are released and develop into tetrasporophytes that produce the meiotic tetraspores (Figure 1).

Recent reviews continue to focus on a plethora of external factors that control algal reproduction such as light (intensity, quality, photoperiod), temperature, season, nutrients (be they inorganic or organic), biotic factors (extracellular algal products, bacterial association, animal grazing), osmotic stress, pH of the medium, wave motion and mechanical shock, pollution, and radiations, and the bulk of knowledge accumulated as to the particular conditions on which these external factors exert their control (Dring, 1988; Bornette and Puijalon, 2011; Agrawal, 2012).

Light and temperature are managed effectively to run the intensive cultivation system of *Porphyra*, but there is a general consensus that increasing our knowledge of the underlying molecular basis of cell growth, development and reproduction in this species, and in economical important seaweeds in general, will improve aquaculture practices (Sahoo et al., 2002; Nakamura et al., 2013).

As we will see below, the same situation occurs when the effect of plant hormones on seaweed reproduction is considered, although some advances at the molecular level have been made on the involvement of certain genes. Almost nothing is known about how the external signals are translated into the molecular mechanisms known to underlie any developmental or reproductive event comprising cell growth and differentiation. Whilst this task was addressed for land plants some time ago, in the genomic era and given the high throughput techniques at our disposal, our knowledge regarding algae in general, and seaweeds in particular, lags far behind that of higher plants and animals. Let us therefore review what is already known about plants and should be revisited in red algae to unveil the secrets of what, no doubt, is also operating at the molecular level to control reproduction.

## REPRODUCTION GENES IN RED ALGAE. DISPARATE MODEL SPECIES AND APPROACHES

As seen in the most recent bibliographic reports, and is evident in this special edition, major advances are taking place in the brown alga since the adoption of *Ectocarpus siliculosus* as the model species and the generalized use of high throughput techniques. This includes key genes in the life cycle transition, developmental pattern, etc. (Peters et al., 2004; Cock et al., 2010; Coelho et al., 2011; Le Bail et al., 2011; Arun et al., 2013). If *E. siliculosus* was chosen mainly because of the particular taxonomic position of brown algae and the evolutionary lineage-related information that could be retrieved from it (Peters et al., 2004), in red algae, no general consensus has been reached as to either the model species or technical approaches and strategies. Consequently, limited advances have been achieved to date, particularly regarding the reproductive event.

*Porphyra* species (Rhodophyta, Bangiophyceae)—or rather *Porphyra/Pyropia* species, as several species has been reassigned as *Pyropia* (Sutherland et al., 2011)—have been proposed as model species, perhaps because of their economic value. The genome and symbiont-free genome have been sequenced (Chan et al., 2012; Nakamura et al., 2013), and 1% of the genes (10,327 total genes predicted) were annotated as related to reproduction in *P. yezoensis*, using the estimation provided by GO Slim in the Blast2Go software (Nakamura et al., 2013). Therefore, this genomic approach always faces a serious constraint, since these genes are commonly assigned to putative biological processes and functions based on the information available for other organisms, that may simply lack genes and functions related to important life cycle or reproduction events in red algae.

The same wide genome approach has recently been used to report the genome and gene annotation of the edible carrageenophyte alga *Chondrus crispus*, the Irish moss (Collén et al., 2013). An important scientific background of knowledge exists for *C. crispus*, to the extent that it has been proposed as the model red alga (Collén et al., 2014). The 9,606 genes annotated for *C. crispus* have produced a very useful bulk of information for the interpretation of the forces driving the evolution of eukaryotic genomes (Collén et al., 2013). Interestingly, *C. crispus* has cryptochromes (Collén et al., 2013), which are important in photosensing and the regulation of the reproduction by light and hormones, as discussed below (Table 1). The phases of the life cycle of *C. crispus* are easily accessible and thus frequently used for experiments, but they might be not so easy to handle if the completion of the life cycle is required, as is the case for reproduction studies, for which mutants are required. Moreover, there is an apparent absence or shortage of well-known key elements in the regulatory network (i.e., absence of phytochromes, phototropins, and a rather small amount of transcription-associated proteins). Therefore, the utility of *C. crispus* as a model species for the study of certain aspects of algal growth and development during life cycle completion (i.e., light control of reproduction) remains a matter for debate.

**TABLE 1:** **An overview of the complex light sensing–plant hormones interaction, highlighting the key molecular factors implicated**.

	**Phytochrome**	**Cryptochrome**	**Phototropins**
Abscisic acid	BBX2, HY5^1^	BBX2, HY5^1^	BBX2, HY5^1^
	PIF1/PIL5^2^	MYC2^?3®^	MYC2^?3®^
		CIB^4,5®^	
Ethylene	PIF3, ERF1^6^	Ethylene synthesis inhibitor^?7^	
	PIF5, DELLA^?7^		
Auxins	PAR, HFR1, PIL1^7^		PKS, 14-3-3 l, PIN, PGP, AUX/LAX^8^
	Gibberellins	DAG, PIF3, PIF4, PIF1/PIL5 COP1, HY5^2^		
Cytokinins	COP1, HY5^2^		
Jasmonic acid	MYC2^?3^		
Brassinosteroids	PIF4, BZR1^9^		

All of the information comes from higher plants, mostly Arabidopsis thaliana. Prominent role are played by transcription factors (TF) and proteins able to interact with them. PIF are a family of bHLH TF able to bind directly to G-BOX in DNA; HY5 is a nuclear constitutive TF; BZR1 is a TF able to bind to PIF4 during brassinosteroids effect on photomorphogenesis; CIB is a bHLH TF that specifically controls Flowering Time locus; MYC2 is a bHLH TF in Arabidopsis; DELLA are proteins that interacts with PIF; COP1 is a ring finger ubiquitin that promotes HY5 degradation; HFR1 is a protein able to interact with PIF or BBX2, a zinc fingers proteins able to repress or modulate the action of transcription factors. The remaining factors are more specific of the plant hormones or the photoreceptor signaling pathways. ® Denotes participation in reproductive events (i.e., flowering). Details on how they interact can be found in the original references (^1^Xu et al., 2014; ^2^Lau and Deng, 2010; ^3^Gupta et al., 2012; ^4^Liu et al., 2013; ^5^Fernando and Coupland, 2012; ^6^Zhong et al., 2012; ^7^Alabadí and Blázquez, 2009; ^8^Hohm et al., 2013; ^9^Jaillais and Grégory, 2012). ? Denotes pending confirmation.

Analysis of the transcriptomes has revealed preferential genes expressed in gametophytes or sporophytes. In *Porphyra purpurea*, an unusual elongation factor (EF-1a) was expressed only in the sporophyte while a second gene, EF, was expressed equally in the sporophyte and the gametophyte (Liu et al., 1996). The PyKPA1 gene, which encoded a sodium pump, was differentially expressed in the gametophyte as compared to the sporophyte, which seems to depend on the presence of specific promoter elements (Uji et al., 2012, 2013). Apart from *Porphyra/Pyropia*, other species also considered to be of economic interest, such as the agarophytic species, have been studied. In this regard, carposporophyte-specific genes were identified in *Gracilariopsis andersonii* (Kamiya et al., 2011). In *Gracilaria lemaneiformis*, a female gametophyte-specific gene, GMF-01, has been reported (Chen et al., 2011) while an ubiquitin gene was also characterized as particularly active during the carposporophyte formation (Ren et al., 2009). In *Griffithsia japonica*, the GjFP-1 gene, encoding a heat-shock protein 90, may be involved in the differentiation of female gametophyte (Lee et al., 1998).

Other approaches have made it possible to reach candidate gene(s) involved in reproduction. This is the case of the GiODC gene in *Grateloupia imbricata*, which encodes the ornithine decarboxylase (ODC, EC. 4.1.1.17). The ODC starts the synthesis of the common polyamines putrescine, spermidine, and spermine by decarboxylating the ornithine to produce putrescine; these substances affect spore maturation and liberation as described below (García-Jiménez et al., 1998; Marián et al., 2000; Guzman-Urióstegui et al., 2002, 2012; Sacramento et al., 2004, 2007). GiODC was cloned using a somewhat laborious approach by means of degenerated primers designed from conserved protein motifs, followed by chromosome walking by iPCR to complete the sequence (García-Jiménez et al., 2009). GiODC expression varied according to cystocarp differentiation with lower levels in the fertile, as compared to the infertile, tissue (García-Jiménez et al., 2009).

All of these findings are clearly contributing to our knowledge about reproduction in red seaweeds, whether achieved through a wide genome strategy using high throughput methods as done in *Porphyra/Pyropia* or *Chondrus*, or using a candidate gene approach as in the case of ODC in *Grateloupia imbricata*. Nevertheless, the weakest point still remains on how this—perhaps species-specific—information can be translated into data that is relevant to most red seaweeds; how to construct a reliable “red seaweed conceptual framework” of knowledge on reproduction from these disparate approaches and species strategies. From our point of view this could only be started to achieve using a species that is easy to handle, with relatively short generation times, and fulfills the criteria needed to undergo genetic transformation, which constitutes the current bottleneck in the molecular biology of seaweeds (see Mikami, 2014).

## LIGHT AND PLANT HORMONES SIGNALING AND INTERACTION. THE WAY TO UNVEIL THE MOLECULAR SECRETS OF RED ALGAL REPRODUCTION?

In photosynthetic eukaryotes, like algae, light is the driving force for growth and development; it is the source of energy, but also the signal triggering both vegetative and reproductive developmental events. Light is perceived through families of photoreceptors: phytochromes (red/far red radiation), UVR8 (UV-B), and membrane associated phototropins, cryptochromes, and the members of the ZTL/FKF1/LKP2 family which absorb UV-A/blue light (Hohm et al., 2013 and references therein). In turn, the existence and the type of photoreceptors in aquatic organisms have attracted scientific attention, due to the peculiar characteristics of the interaction of light in the aquatic environment. Thus, the presence of diverse genuine photoreceptors, such as phototropins, aurochromes (blue absorbing), neochromes—a kind of chimeric phytochrome, cryptochromes, and phytochromes in marine algae has been reported and reviews have been produced, which include future applied dimensions (Kianianmomeni and Hallmann, 2014). Interestingly, as far as signal transduction is concerned, the phototropin mechanism seems to be conserved between algae and higher plant (Huang et al., 2002; Onodera et al., 2005; Prochnik et al., 2010).

In seaweeds, plant hormones have been reported to affect growth and development (Chan et al., 2006; Baweja et al., 2009). Concerning reproduction events, in *Grateloupia imbricata* (as *G. doryphora*), the levels of the polyamines putrescine, spermidine, and spermine changed as the cystocarps maturate. Subsequently, it was observed that these polyamines, particularly spermine, favored the maturation, liberation, and growth of carpospores in *Grateloupia imbricata* and *Hydropuntia cornea* (as *Gracilaria cornea*; García-Jiménez et al., 1998; Marián et al., 2000; Guzman-Urióstegui et al., 2002, 2012; Sacramento et al., 2004, 2007). In addition, ethylene has been reported to accelerate the maturation of tetrasporangia in *Pterocladiella capillacea* (García-Jiménez and Robaina, 2012)

In recent years extensive knowledge has been accumulated about light, photoreceptors and plant hormone interaction, and crosstalk at the molecular level in higher plants, particularly during the events occurring at two physiological scenarios: seedling photomorphogenesis and shade avoidance (Gyula et al., 2003; Lau and Deng, 2010). Transcription factors of several families, protein–protein interaction, as well as post-translational protein modification are involved (Alabadí and Blázquez, 2009; Lau and Deng, 2010; Fernando and Coupland, 2012; Gupta et al., 2012; Jaillais and Grégory, 2012; Zhong et al., 2012; Liu et al., 2013). Table 1 provides an overview of the complex system operating in the light–photoreceptors–plant hormones integrated network. All of this important information has so far proved to be relevant to higher plants, particularly for the model species *Arabidopsis thaliana*, and it is completely unknown whether the key elements highlighted also operate in seaweeds, despite the fact that the influence of photoreceptors and plant hormones on reproduction has been reported, as previously mentioned.

Transcription factors controlled by photoreceptors, such as the PIF family (Alabadí and Blázquez, 2009; Lau and Deng, 2010; Jaillais and Grégory, 2012; Zhong et al., 2012), along with others under the control of plant hormones, such as HY5 (Lau and Deng, 2010; Xu et al., 2014), are very important players in the integrated network (Table 1). Other factors affecting plant growth and development, like circadian clock sensors that control endogenous levels of plant hormones (i.e., auxin), or temperature also seem to act by modulating the activity of PIF transcription factors, thus connecting important abiotic factors and development (Leivar and Quail, 2011).

Finally, by way of future perspective, in our laboratory we have recently adopted *Bonnemaisonia hamifera* (Bonnemaisoniaceae) as a working species. Cultures of the sporophyte (*Trailliella*) phase have been established so far, and work is progressing toward the induction of the differentiation of gametophytes, using temperature and photoperiod, and plant hormones (Figures 1A–C). Should the completion of the life cycle may be accomplished in the next future as a basic requirement for a working species, it remains to find an appropriate genomic structure (a compact or simple genome, a small number of genes, important functional transcriptomic information, etc.), but there is little information on the *B. hamifera* genome so far. Nevertheless, even in the long run, with *B. hamifera* or any other similar and more adequate species, it is time to revisit this scenario in seaweeds but focusing on the molecular standpoint; why not start identifying within the rising genomic/transcriptomic data for all or any of these regulating factors shown in Table 1?

### Conflict of Interest Statement

The authors declare that the research was conducted in the absence of any commercial or financial relationships that could be construed as a potential conflict of interest.
